# MicroRNA-214 Suppresses Osteogenic Differentiation of Human Periodontal Ligament Stem Cells by Targeting ATF4

**DOI:** 10.1155/2017/3028647

**Published:** 2017-10-29

**Authors:** Siqi Yao, Wei Zhao, Qianmin Ou, Lanchen Liang, Xuefeng Lin, Yan Wang

**Affiliations:** Guanghua School of Stomatology, Guangdong Provincial Key Laboratory of Stomatology, Sun Yat-sen University, Guangzhou 510055, China

## Abstract

Periodontitis is the main cause of adult tooth loss. Stem cell-based tissue engineering has become a promising therapy for periodontitis treatment. To date, human periodontal ligament stem cells (hPDLSCs) have been shown to be a favorable source for tissue engineering, but modulatory mechanisms of hPDLSCs remain unclear. Approximately 60% of mammalian genes are the targets of over 2000 miRNAs in multiple human cell types, and miRNAs are able to influence various biological processes in the human body, including bone formation. In this study, we found that after osteogenic induction, miR-214 was significantly decreased in hPDLSCs; therefore, we examined the effects of miR-214 on osteogenic differentiation. Computational miRNA target prediction analyses and luciferase reporter assays revealed that activating transcription factor 4 (ATF4) is a direct target of miR-214. We prepared cells overexpressing miR-214 and found that miR-214 negatively regulates osteogenic differentiation of hPDLSCs. For the target of miR-214, ATF4 protein expression level was decreased after induction. In conclusion, we found that miR-214-ATF4 axis is a novel pathway for regulating hPDLSC osteogenic differentiation.

## 1. Introduction

Periodontitis is one of the most widespread infectious diseases, and the main cause of adult tooth loss, and even matter with some high prevalence diseases such as diabetes, pulmonary infections, and chronic kidney diseases [1–4]. Although current periodontal therapies are incapable of regenerating normal structure and functionality, stem cell-based tissue engineering offers a promising strategy for the regeneration of lost or damaged periodontal tissue [5, 6]. Human periodontal ligament stem cells (hPDLSCs) are more accessible than human bone marrow stem cells and possess great potential as a source for tissue engineering [7–11]. hPDLSCs were shown to possess superior abilities for differentiating into bone, cementum, and periodontal ligament [11–13]. A clinical autologous hPDLSC treatment for cell-based periodontal therapy was shown to be safe and effective [14], but modulating the biological functions of hPDLSCs requires further examination.

MicroRNAs (miRNAs) are single-stranded noncoding RNAs that bind complementary sequences of target mRNAs in the 3′ untranslated regions (3′UTRs). They alter the levels of gene expression after transcription in various cellular processes [15, 16] since approximately 60% of mammalian genes are the targets of over 2000 miRNAs in multiple types of human cells [17]. miRNAs contribute to various steps of bone remodeling [18, 19], and various miRNAs have been demonstrated to regulate the complicated processes of osteogenic differentiation in hPDLSCs [20–25].

Numerous studies have examined the role of miR-214 in bone formation [26–28]. One study demonstrated that miR-214 targeted Osterix to inhibit osteogenic differentiation in C2C12 myoblast cells [26]. One study showed that miR-214 inhibited osteoblast activity by targeting activating transcription factor 4 (ATF4) [27]. Another study suggested that osteoblast activity was suppressed by exosomal miR-214-3p derived from osteoclast *in vitro* and bone formation was inhibited *in vivo* [28]. However, it remains unclear whether the expression of miR-214 changes after osteogenic induction in hPDLSCs and, if so, what its regulation function may be.

In this study, we found changes in the miR-214 expression levels before and after osteogenic induction in hPDLSCs and used miRbase bioinformatic analysis to predict the direct target of miR-214. Among the candidates, ATF4 was selected for further analysis. This study found a regulatory role for miR-214 in the osteogenic differentiation of hPDLSCs by targeting ATF4.

## 2. Materials and Methods

### 2.1. Isolation of hPDLSCs and Cell Culture

Human periodontal ligament tissues were obtained from premolars from 20 donors (aged 13–24 years, 10 men and 10 women, without oral or systematic diseases) during orthodontic procedures under informed consent. Ethics Committee approval was provided by the School of Stomatology, Sun Yat-sen University.

hPDLCs were harvested and cultured as previously reported [29]. Tissues attached to the middle third of tooth root was collected and cut into small pieces (1 mm^3^) followed by digestion with 3 mg/mL type 1 collagenase (Life Technologies, Carlsbad, CA, USA) and 4 mg/mL dispase (Life Technologies) at 37°C for 1 h. The tissues were cultured in complete *α*-minimum essential medium (Gibco, Grand Island, NY, USA) containing 10% (*v*/*v*) fetal bovine serum (Gibco), 100 U/mL penicillin, 100 *μ*g/mL streptomycin (HyClone, Logan, UT, USA), and 5 mM L-glutamine (Gibco). The medium was changed every 3 days. When the human periodontal ligament cells reached 80% confluence, they were subcultured.

### 2.2. Colony-Forming Efficiency Assessment

Colony-forming efficiency was assessed as previously reported [29]. Briefly, hPDLSCs were seeded into 10 cm culture dishes at a density of 1 × 10^3^ cells per dish. After culturing for 12 days, 4% paraformaldehyde was added to the dishes for 20 min to fix the cells, followed by staining with 0.1% (*w*/*v*) crystal violet (Sigma-Aldrich, St. Louis, MO, USA). Aggregates of 50 or more cells were counted as a colony unit.

### 2.3. Immunofluorescence Staining

hPDLSCs were fixed with 4% paraformaldehyde in phosphate-buffered saline (PBS) for 20 min, washed with PBS three times, and blocked with 5% bovine serum albumin/ PBS for 1 h. hPDLSCs were incubated with primary antibodies (mouse antivimentin and mouse anticytokeratin, 1 : 100; Life Technologies) overnight at 4°C. The cells were washed with PBS three times and then incubated with goat anti-mouse IgG antibody (1 : 300; Alexa Fluor 488; Life Technologies) for 45 min at 25°C in the dark. After staining with Hoechst 33342 (Life Technologies), images were recorded using a Zeiss Axio Observer Z1 (Cael Zeiss, Oberkochen, Germany).

### 2.4. Surface Marker Expression

A total of 5 × 10^5^ cells was collected and washed three times with PBS and then incubated with the antibodies of PE-labeled anti-CD44, anti-CD90, anti-CD105, anti-CD166, anti-CD34, anti-CD45, and anti-HLA-DR (1 : 10; BD Biosciences, Franklin Lakes, NJ, USA). Marker fluorescence was detected immediately using a BD Accuri C6 (BD Biosciences). Data were analyzed using CF Low Plus Software (BD Biosciences).

### 2.5. Osteoblast and Adipocyte Differentiation

For osteoblastic differentiation, hPDLSCs were seeded in 6-well plates at a density of 1 × 10^5^ per well. After cell adhesion, the culture medium was replaced with osteogenic medium containing 10% fetal bovine serum, 10 mM *β*-glycerophosphate, 10 nM dexamethasone, and 50 *μ*g/mL ascorbic acid. The osteogenic medium was changed every 3 days.

For adipocyte differentiation, hPDLSCs were seeded as described above. Cells were cultured in adipogenic medium (Cyagen Biosciences, Santa Clara, CA, USA). Two weeks later, the cells were fixed as described above. Oil red O solution was added and incubated for 15 min and then washed extensively with PBS. The images were recorded.

### 2.6. Alizarin Red Staining

hPDLSCs and hPDLSCs-miR-214/hPDLSCs-control were collected and fixed using 4% paraformaldehyde after 21 days of culture in osteogenic medium and then incubated with 40 mM Alizarin red S (Sigma) for 15 min with gentle shaking. The cells were washed three times to remove the unstained color with PBS. The red positions were recognized as mineralized nodules. After images were recorded, nodules were dissolved using 10% (*w*/*v*) cetylpyridinium chloride (Sigma-Aldrich), and absorbance was evaluated spectrophotometrically at 562 nm.

### 2.7. Alkaline Phosphatase Activity Assay

hPDLSCs-miR-214/hPDLSCs-control was lysed after 7 and 14 days of culture in osteogenic medium, and the alkaline phosphatase (ALP) activity of the groups was measured using an ALP assay kit (Beyotime Biotech, Shanghai, China) according to the manufacturer's instructions. Absorbance was evaluated spectrophotometrically at 405 nm.

### 2.8. Transfection of hsa-miR-214 Inhibitor (Inhibitor-214)

Inhibitor-214 (micrOFF™ miRNA inhibitor, Ribobio, Guangzhou, China) was transfected into hPDLSCs by Lipofectamine LTX (Life Technologies) according to the manufacturer's protocol. The medium was changed to osteogenic medium after 24 h.

### 2.9. Quantitative Real-Time Reverse Transcription Polymerase Chain Reaction (qRT-PCR)

Total RNA was isolated from cells using Trizol (Life Technologies), and the concentration was determined by spectrophotometry. For mRNA quantification, 1 *μ*g of total RNA was reverse-transcribed to cDNA using the Reverse Transcriptase M-MLV Kit (Takara, Shiga, Japan). Gene expression was quantified by qPCR with a SYBR Green kit (Roche, Basel, Switzerland) and gene-specific primers. Amplification conditions were set as follows: 95°C for 10 min, 40 cycles of denaturation at 95°C for 15 s, annealing at 60°C for 20 s, and final extension at 72°C for 20 s. The GAPDH gene was used to normalize the mRNA data. Next, 1 *μ*g of total RNA was reverse-transcribed using Bulge-LoopTM miRNA RT Primer (Ribobio) specific for human miR-214, and qPCR was performed on a Roche Light Cycler System using Bulge-Loop miRNA qRT-PCR starter kit (Ribobio). Human small nuclear RNA U6 was used to normalize mature miRNA data. For microRNA absolute quantification, miDETECT™ miRNA qRT-PCR Standard RNA of miR-214 and U6 (Ribobio) was used to create standard curves following the manufacturer's protocol, and copy numbers were calculated according to the standard curves. The sequences of primers are listed in Table 1.

### 2.10. Prediction of Target Gene

We conducted computational miRNA target prediction analyses to identify potential targets, including the programs miRanda, PicTar, and TargetScan. Among the candidates, a miR-214 binding site was detected in the 3′UTR of ATF4.

### 2.11. Plasmid Construction

The 3′UTR of ATF4 was produced by PCR with primers (5′-TCGAAGATCTTTTCTACTTTG-CCCG-3′ and 5′-TGGCGAGCTCTACTTTCCCTACAAA-3′) inserted into the pMIR-REPORT-luciferase vector (provided by Dr. Yan Yuan, University of Pennsylvania) and named as ATF4-3′UTR-WT. A suitable mutant type (with seed sequence deletion) was prepared using primers (5′-CGCGACGCGTGATAGTCAGGAGCGTCAATG-3′ and 5′-AGCTTTGTTTAAACACTTTC-CCTACAAAATAAT-3′) and inserted into the same report vector and named as ATF4-3′UTR-Mut.

A miR-214 minigene was produced by PCR with primers (5′-GCGCGGATCCTTTTCTCCCTT-TCCCCTTACTCT-3′ and 5′-CCGGAATTCCGAGCCCCTCATTTTGGTTGTAG-3′) and inserted into the pSIF-neo-IRES-GFP plasmid (provided by Dr. Yan Yuan, University of Pennsylvania) between EcoRI and BamHI sites; this plasmid was referred to as pSIF-miR-214. The empty plasmid served as a control.

### 2.12. Luciferase Reporter Assay

HEK-293T cells were cotransfected with hsa-miR-214 mimic (mimic-214, micrON™ miRNA mimic, Ribobio) and ATF4-3′UTR-WT/Mut (and their controls) by Lipofectamine LTX (Life Technologies) according to the manufacturer's protocol. pRL-TK renilla served as the luciferase internal control. The activity of luciferase/renilla was evaluated at 36 h posttransfection using GloMax 96 Microplate Luminometer (Promega, Madison, WI, USA). The mean value of the group of cells transfected with controls was confirmed as 1.0.

### 2.13. Establishment of Stable Cell Line

First, 4.5 *μ*g pSIF/pSIF-miR-214 microRNA expression vector or control vector, 4.5 *μ*g pFIV-34N lentiviral gag-pol packaging vector, and 0.57 *μ*g pVSV-G envelope vector were transfected into HEK293T cells using the calcium phosphate transfection method. Three days later, lentiviral particles were gathered and filtered through a 0.45 *μ*m filter. hPDLSCs were infected with lentivirus and spinoculated at 2500 rpm for 1 h at room temperature with 8 *μ*g/mL polybrene and then incubated in a 5% CO_2_ incubator at 37°C for 4 h. The inocula were then removed and replaced with fresh media. At 48 h postinfection, cells were selected by G418 treatment. Successfully infected cells were named as hPDLSCs-miR-214, while control cells were named as hPDLSCs-control.

### 2.14. Western Blotting

RIPA buffer (50 mM Tris-HCl pH 7.5, 150 mM NaCl, 1% NP-40, 0.5% sodium deoxycholate, and 0.1% SDS with complete protease inhibitor cocktail) was used for cell lysis. Total protein concentrations were evaluated using a protein assay kit (Pierce). Total protein (40 *μ*g) was separated by electrophoresis on 8% sodium dodecyl sulfate polyacrylamide gels and then transferred to a nitrocellulose membrane. Subsequently, the membranes were blocked for 1 h at room temperature by incubation in PBS containing 5% (*w*/*v*) nonfat milk and 0.1% (*v*/*v*) Tween-20 followed by overnight incubation in the blocking buffer at 4°C with primary antibodies against ATF4 (Sigma), RUNX2 (Boster Bio, Pleasanton, CA, USA), and *β*-actin (Sigma). Finally, the membranes were incubated with secondary antibodies for 30 min and analyzed with an Odyssey two-color infrared laser imaging system (LI-COR Biosciences, Lincoln, NE, USA). Three separate experiments were performed. The relative density of labeled protein bands was analyzed with Image-ProPlus 5.0 software (Media Cybernetics Inc., Rockville, MD, USA).

### 2.15. Statistics

All data are presented as the mean ± standard error from at least three independent experiments. The SPSS 20.0 software package (SPSS Inc., Chicago, IL, USA) was used for the statistical tests. Student's *t*-test was used for comparison among groups. In this study, *p* < 0.05 was considered to indicate statistical significance.

## 3. Results

### 3.1. Characterization of hPDLSCs

**Figure 1:**
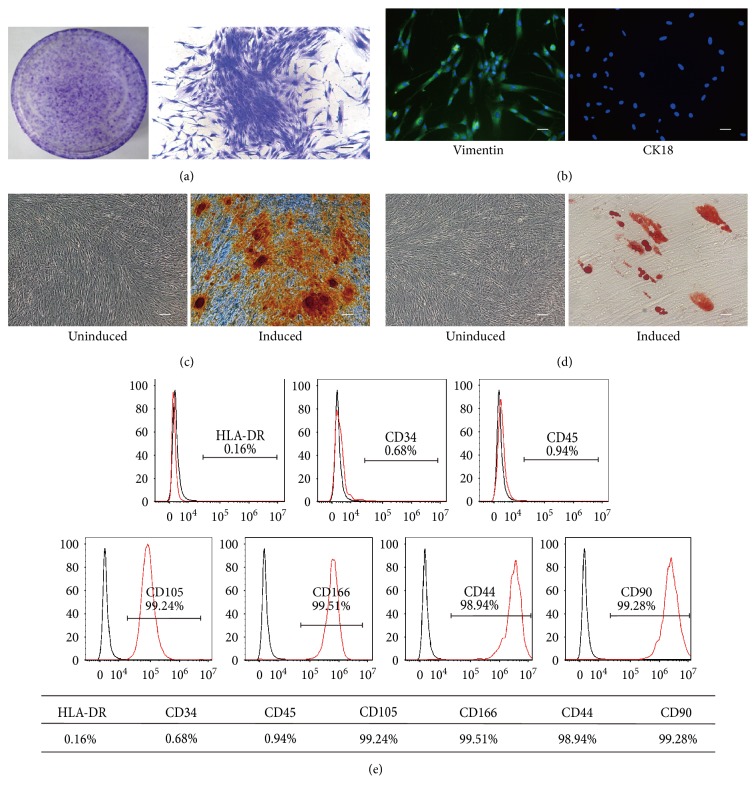
Characteristics of human periodontal ligament stem cells (hPDLSCs). (a) Representative images of a single colony-forming unit of hPDLSCs at 12 days. (b) Immunocytochemical staining of the 3rd passage of hPDLSCs expressing vimentin but not CK18. (c-d) Multiple differentiation ability of hPDLSCs. hPDLSCs demonstrated by Alizarin red (c) and (d) Oil red O staining under specific differentiation conditions for osteoblasts or adipocytes. (e) Cell surface markers of hPDLSCs were detected by flow cytometry. Scale bar, 100 *μ*m.

hPDLSCs were collected from the root surface of healthy premolars and mixed to decrease individual differences (Figure S1a available online at https://doi.org/10.1155/2017/3028647). The cells showed plastic-adherent characteristic and clearly formed colonies after 12 days of culture (Figure 1(a)). Immunocytochemical staining was used to detect markers for mesenchymal cells and epithelial cells. Cells subcultured to passage 3 showed positive expression of vimentin, a marker of mesenchymal stem cells (MSCs), and negatively expressed CK18, an epithelium marker (Figure 1(b)). Next, hPDLSCs were cultured in osteogenic and adipogenic induction medium for 21 days. These cells exhibited Alizarin red-positive calcium deposition (Figure 1(c)) and Oil red O-positive lipid accumulation in the cytoplasm (Figure 1(d)), and adipogenesis was confirmed by qRT-PCR (Figure S1b). We detected surface markers of hPDLSCs by flow cytometry. Our results demonstrated that hPDLSCs express the markers of MSCs, including CD105 (99.24%), CD166 (99.51%), CD44 (98.94%), and CD90 (99.28%), and were negative for the cell markers CD34 (0.68%), CD45 (0.94%), and HLA-DR (0.16%) (Figure 1(e)). Taken together, these results demonstrate that isolated hPDLSCs are stem cells of mesenchymal origin with powerful multipotency.

### 3.2. miR-214 Is Downregulated after Osteogenic Induction

**Figure 2:**
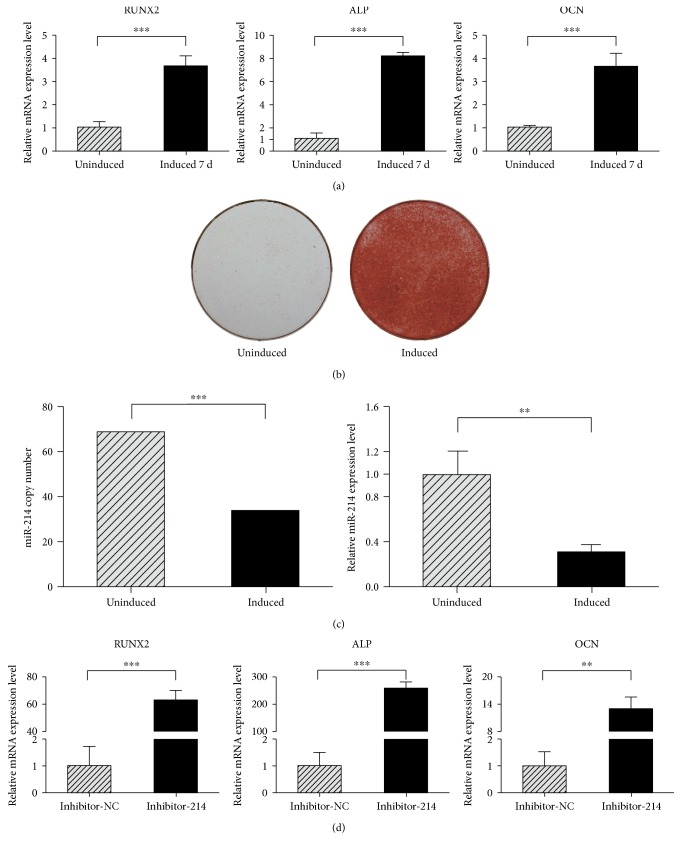
miR-214 expression decreased after osteogenic induction. (a) Osteogenic induction was verified by increased expression of RUNX2, ALP, and OCN in mRNA level. (b) Alizarin red staining was performed at 21 days. (c) qRT-PCR was used to quantify miR-214 absolute (left) and relative (right) expression levels after 7 days of osteogenic differentiation. Absolute quantitation was normalized to 100 U6 copies. (d) After transfection of inhibitor-214 for 72 h, qRT-PCR was conducted to detect osteogenesis gene markers. ^∗∗^*p* < 0.01 and ^∗∗∗^*p* < 0.001.

To evaluate the differences in miRNA expression during osteogenesis in hPDLSCs, we performed miRNA array assays (unpublished data). We found that during osteogenic differentiation, miR-214 was significantly downregulated. To confirm the array results, hPDLCSs were osteogenic-induced in vitro, and induction was observed as the strong expression level of RUNX2, ALP, and OCN (Figure 2(a)). Alizarin red staining was conducted on day 21 to visualize the characteristic mineralized nodules (Figure 2(b)). We also assessed the absolute and relative expression level of miR-214 by qRT-PCR, which was reduced after induction (Figure 2(c)). The results showed that the expression level of miR-214 was decreased after osteogenic induction. Next, we evaluated the influence of transfection with inhibitor-214 in hPDLSCs, and the osteogenic marker genes such as RUNX2, ALP, and OCN showed stronger expression levels after inhibited miR-214 (Figure 2(d)). These data indicate that after osteogenic induction, miR-214 expression level decreased markedly, which agreed with the results of our miRNA array analysis.

### 3.3. Identification of miR-214 Target

**Figure 3:**
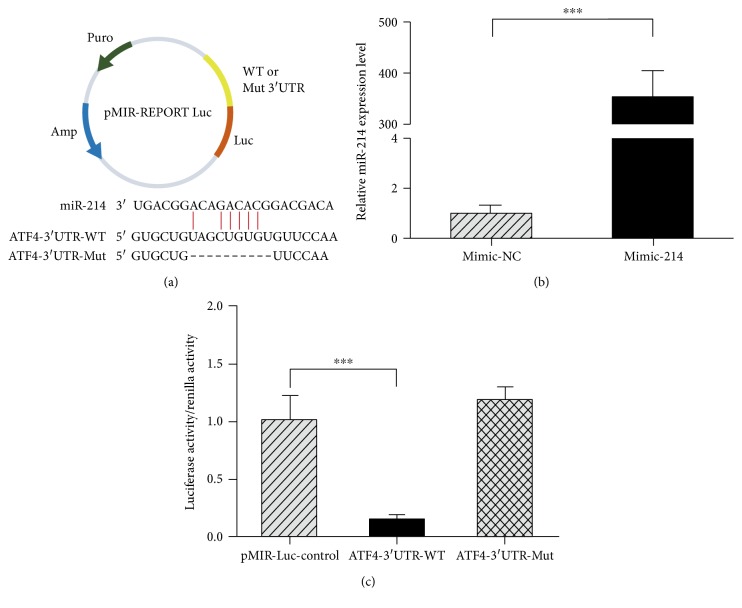
miR-214 directly targets ATF4. (a) Diagram of luciferase reporters, wild-type ATF4 3′UTR (WT), or mutant ATF4 3′UTR (Mut). (b) HEK-293T cells were transfected with mimic-214 and qPCR was performed after 36 h. (c) The effect of mimic-214 with ATF4-3′UTR-WT/Mut on luciferase activity in HEK-293T cells. The luciferase/renilla activity of the groups was calculated. ^∗∗∗^*p* < 0.001.

To gain further insight into how miR-214 influences the osteogenic differentiation of hPDLSCs, computational miRNA target prediction analyses were conducted. This analysis revealed a potential miR-214 binding site in 3′UTR of ATF4. To confirm that ATF4 binds miR-214 at this site, the 3′UTR containing the recognition sequence of ATF4 was cloned into a luciferase reporter vector (pMIR), and the suitable mutant type with its seed sequence deletion was inserted into the same reporter vector (Figure 3(a)). Mimic-214, a chemically modified double-stranded RNA that mimics endogenous miR-214, was transfected into HEK293T cells, and qPCR was performed at 36 h posttransfection. The miRNA-214 level was approximately 340-fold higher in mimic-214-transfected cells than in mimic-NC-transfected cells (Figure 3(b)). We cotransfected the reporter ATF4-3′UTR-WT with mimic-214 into HEK293T cells, then luciferase activity and renilla activity were measured. The results showed that the activity of group ATF4-3′UTR-WT was significantly lower than that in the control (Figure 3(c)). Furthermore, when the miR-214 target site was deleted from the ATF4 3′UTR reporter vector (ATF4-3′UTR-Mut), the activity was restored and the reporter was no longer responsive to mimic-214 (Figure 3(c)). These results suggest that miR-214 directly targets ATF4 through the predicted recognition sequence in the 3′UTR.

### 3.4. miR-214 Downregulates ATF4 Protein Expression

**Figure 4:**
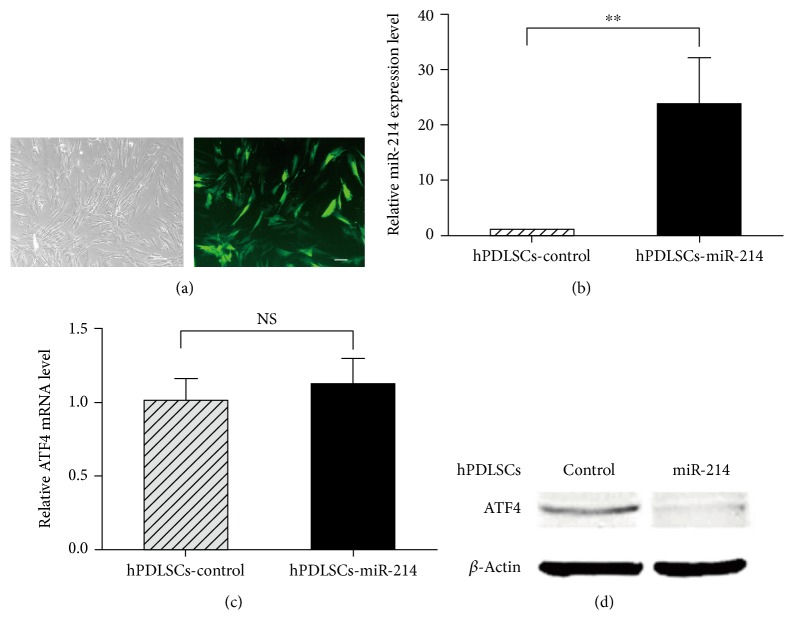
miR-214 downregulates ATF4 protein expression. (a) Stable cell line hPDLSCs-miR-214 overexpressing miR-214 was constructed by lentivirus marked with GFP. Representative image of hPDLSCs-miR-214. Scale bar, 100 *μ*m. (b) qPCR analysis was performed to detect miR-214 expression within hPDLSCs-miR-214. (c, d) After 14 days of culture, qRT-PCR and Western blotting were conducted to determine the mRNA and protein levels of ATF4. ^∗∗^*p* < 0.01; NS: no significance.

To gain a better understanding of the endogenous relationship between miR-214 and ATF4, we constructed plasmid pSIF-miR-214 to establish a stable cell line overexpressing miR-214, referred to as hPDLSCs-miR-214 (Figure 4(a)); an empty vector was used as a control (hPDLSCs-control). The miR-214 expression level in hPDLSCs-miR-214 was confirmed by real-time PCR, and the result demonstrated that miR-214 expression was higher by approximately 24-fold in hPDLSCs-miR-214 than that in control cells (Figure 4(b)).

To confirm whether miR-214 indeed downregulates ATF4 expression, we examined the ATF4 expression level in hPDLSCs-miR-214 by qPCR and Western blot analysis. Interestingly, gene expression analysis revealed no significant differences in ATF4 mRNA level (Figure 4(c)), while Western blot analysis showed decreased ATF4 protein level when miR-214 was overexpressed in hPDLSCs (Figure 4(d)). Taken together, these findings suggest that miR-214 regulates ATF4 via modification of protein synthesis.

### 3.5. miR-214 Suppresses Osteogenic Differentiation

**Figure 5:**
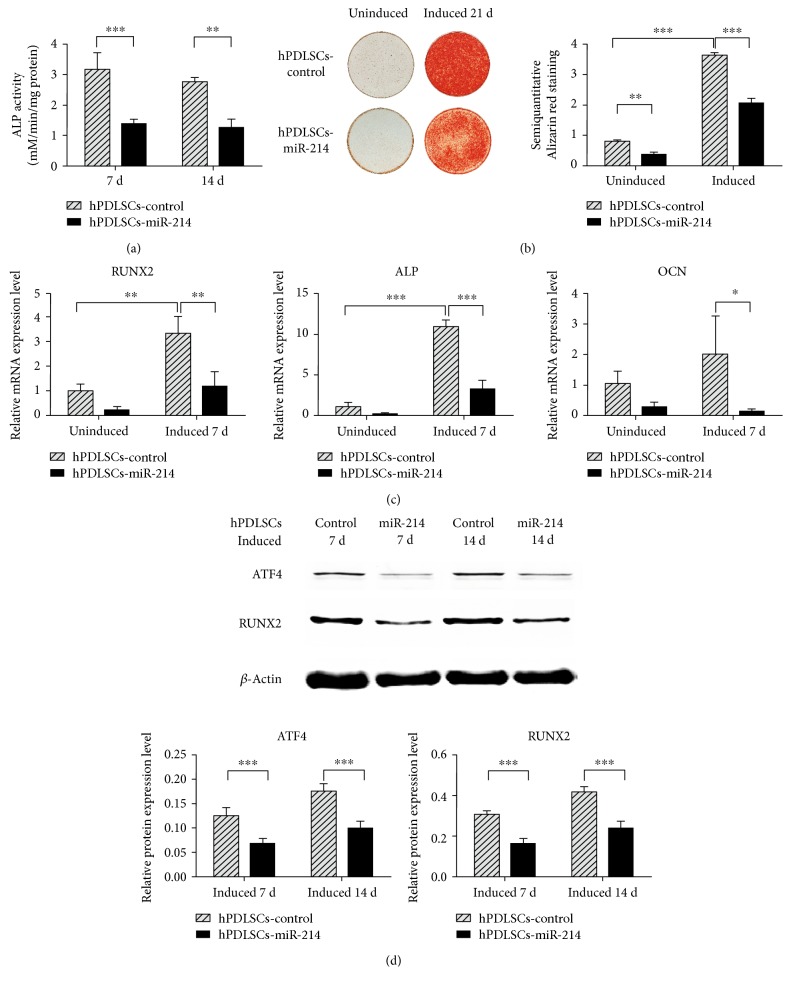
miR-214 negatively regulates osteogenic differentiation of hPDLSCs. (a) ALP activity assay was measured at 7 and 14 days. (b) Alizarin red staining of calcium deposition in hPDLSCs-miR-214 and hPDLSCs-control with or without osteogenic induction (left) and the semiquantitative results (right). (c) Osteogenic marker genes RUNX2, ALP, and OCN were evaluated by qPCR after 7 days osteogenic induction. (d) ATF4 and RUNX2 protein in hPDLSCs-miR-214 and hPDLSCs-control were evaluated by western blot analysis after induction for 7 and 14 days. ^∗^*p* < 0.05, ^∗∗^*p* < 0.01, and ^∗∗∗^*p* < 0.001.

To explore the function of miR-214 in osteogenic differentiation of hPDLSCs, we assayed the ALP activity of hPDLSCs-miR-214 at different time points. These results showed that ALP activity was remarkably lower than in controls (Figure 5(a)). Alizarin red staining after 21 days of osteogenic induction also showed that miR-214 markedly suppressed osteogenic differentiation (Figure 5(b)). The mRNA levels of the osteogenic marker genes RUNX2, ALP, and OCN were measured in hPDLSCs-miR-214 and hPDLSCs-control by qPCR after 7 days of osteogenic induction. Notably, overexpression of miR-214 apparently suppressed the expression level of these marker genes (Figure 5(c)). Western blotting showed similar outcomes, with RUNX2 expression level decreased significantly in hPDLSCs-miR-214 at both 7 and 14 days (Figure 5(d)). As the target gene of miR-214, ATF4 showed a decreased protein level (Figure 5(d)). Based on these findings, miR-214 negatively regulates the osteogenic differentiation of hPDLSCs.

### 3.6. miR-214 Promotes Adipogenic Differentiation

**Figure 6:**
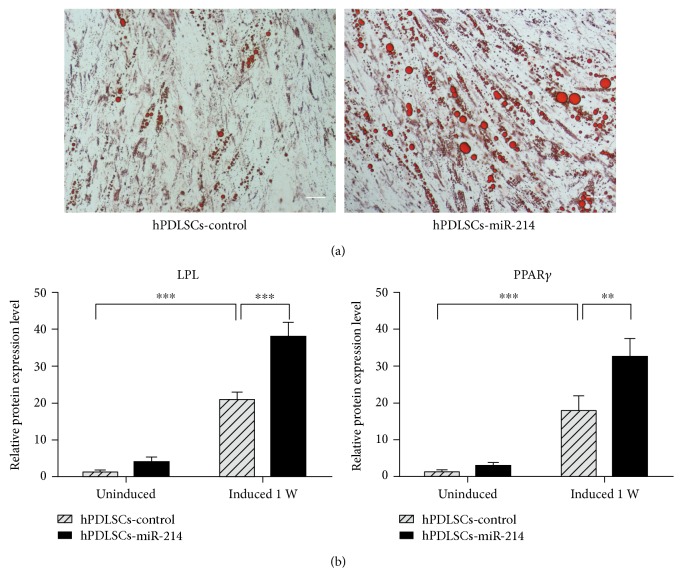
miR-214 promotes adipogenic differentiation of hPDLSCs. (a) Oil red O staining of hPDLSCs-miR-214 and hPDLSCs-control after 2 weeks induction. Scale bar, 50 *μ*m. (b) Adipogenic marker genes LPL and PPAR*γ* were evaluated by qRT-PCR after 1 week adipogenic induction. ^∗∗^*p* < 0.01 and ^∗∗∗^*p* < 0.001.

To confirm the function of miR-214 in adipogenesis, hPDLSCs-miR-214 was adipogenic-induced, and Oil red O staining and qRT-PCR were performed. The staining results showed that at 2 weeks after induction, cells overexpressing miR-214 showed stronger adipogenesis (Figure 6(a)). The qRT-PCR results also showed the same trend in the expression level of adipogenic marker genes LPL and PPAR*γ* at 1 week after induction (Figure 6(b)).

## 4. Discussion

In recent years, stem cell-based periodontal therapy exhibits promising therapeutic potential, in which stem cells are an important part apparently. So the sort of stem cells becomes one of the central issues in stem-cell based periodontal therapy. In the early works, the potential of human bone marrow mesenchymal stem cells (hBMMSCs) has been widely investigated and hBMMSCs were proved that they do regenerate periodontal tissues to some extent [30–36].

hPDLSCs are a population of postnatal stem cells with the potential to differentiate into osteoblasts, odontoblasts, adipocytes, and neuronal-like cells [11, 37, 38]. Compared to hBMMSCs, hPDLSCs appeared to be more advantageous. Firstly, hPDLSCs are more accessible, as they can be collected during orthodontic procedures or from the third molar while hBMMSCs must be collected from bone marrow, requiring additional trauma to the patient. Secondly, hPDLSCs have a higher proliferation rate and levels of specific transcription factors than hBMMSCs [11, 39] and other types of dental stem cells such as dental pulp stem cells and periapical follicular stem cells [11, 12, 39]. Thirdly, as our main goal was to regenerate periodontal structures, hPDLSCs would be a preferred choice because hPDLSCs form cementum-like and periodontal ligament-like structures in immunocompromised mice after transplantation [11], while other types of mesenchymal stem cells have not been reported to have this ability. Fourthly, hPDLSCs can restrict inflammatory reactions and immune responses [40], which is remarkable because most clinical patients who require periodontal regeneration are under inflammatory conditions. Based on these unique characteristics, hPDLSCs are considered as one of the best candidates for future dental clinical applications. This is the first study to reveal that the miR-214-ATF4 axis is a novel pathway involved in regulating hPDLSC osteogenic differentiation.

Previous studies showed that miR-214 is involved in the osteoblast differentiation of mesenchymal stem cells [27, 28, 41–43], but whether miR-214 affects osteoblast differentiation of hPDLSCs remained unclear. In this study, we found that miR-214 suppressed osteogenesis in hPDLSCs and thus is a potential target for osteoregenerative therapy. In vitro, miR-214 expression was decreased during osteogenesis of hPDLSCs (Figure 2(c)). Transfection of inhibitor-214 made hPDLSCs show higher mRNA expression levels of osteogenic marker genes (Figure 2(d)). Accordingly, computational miRNA target prediction analysis suggested a potential miR-214 binding site in the 3′UTR of ATF4; therefore, ATF4 was further analyzed. This study postulated a regulatory role for miR-214 in the osteogenic differentiation of hPDLSCs by targeting ATF4. The results of luciferase reporter assays showed that miR-214 led to significantly lower luciferase activity in the 3′UTR-ATF4 WT group than that in the controls. When the miR-214 target site was deleted from the 3′UTR-ATF4 reporter vector, luciferase reporter activity was restored (Figure 3(c)). These suggest that miR-214 directly targets the ATF4 3′UTR.

Next, we constructed the plasmid pSIF-miR-214 to develop a stable cell line named as hPDLSCs-miR-214. Cells overexpressing miR-214 were cultured and osteogenically induced to evaluate the expression change of ATF4. The results revealed no change between hPDLSCs-miR-214 and hPDLSCs-control at the ATF4 mRNA level, but protein level of hPDLSCs-miR-214 was significantly reduced (Figure 4(c)), which is similar to the results observed in osteoblasts [27, 28]. Protein processes of ATF4 follow transcriptional, translational, and posttranslational mechanisms [44, 45]; therefore, these data suggest that miR-214 posttranscriptionally modulates the gene expression of ATF4. We measured the mRNA levels of the osteogenic marker genes RUNX2, ALP, and OCN in hPDLSCs-miR-214 and found that expressions were significantly decreased. Western blotting, ALP activity assay, and Alizarin red staining assays showed similar outcomes (Figure 5). Here, we found that after osteoblast induction, miR-214 acts as a suppressor in bone formation of hPDLSCs. As adipogenic differentiation is an important part of hPDLSC differentiation, we also assessed the adipogenesis of hPDLSCs-miR-214. We found that overexpression of miR-214 promoted adipocyte differentiation according to the results of qRT-PCR and Oil red O staining (Figure 6). A previous study showed the same trend in hBMMSCs [43]. Several studies demonstrated that ATF4 is involved in lipid metabolism [46–49]. Overexpression of ATF4 promoted adipogenesis and lipogenesis [49], and ATF4 depletion decreased adipocyte differentiation [46]. Our study seems to obtain a different result from the others, but because a microRNA could target different genes, and one gene could be regulated by different microRNAs, overexpressing miR-214 may activate other pathways to promote the adipogenesis of hPDLSCs, which should be further studied.

To date, various miRNAs have been demonstrated to contribute to the processes of osteogenic differentiation in hPDLSCs. miR-214 is encoded within a larger noncoding RNA, Dnm3 opposite strand (Dnm3os) [50]. miR-214 plays an important role in cancer networks [51] and functions in cell self-renewal by directly targeting catenin beta interaction protein 1 and a number of Wnt signaling pathway molecules [52]. Recently, an increased number of studies have reported that miR-214 also participates in the regulation of osteoblast function [26–28] and osteoclastogenesis [42]. Our data suggest that miR-214 negatively regulates osteogenesis in hPDLSCs.

It has been reported that various signaling proteins and transcription factors regulate the differentiation of hPDLSCs. ATF4 is an important leucine-zipper transcription factor in the cAMP response element-binding protein/ATF family. ATF4 was reported to suppress insulin sensitivity and insulin secretion in osteoblasts by favoring Esp [53] or cooperating with FOXO1 [54] to decrease the activity of osteocalcin. It was reported that miR-214 suppresses glucose production in an ATF4-dependent manner in vivo, and researchers suggested that miR-214 may also regulate gluconeogenesis via AMP-activated protein kinase [55]. In general, ATF4 has been proved to be a vital regulator of insulin sensitivity and the target of miR-214. So it is reasonable to speculate that miR-214-ATF4 axis is involved in regulating insulin sensitivity; further studies are needed to confirm this. Additionally, for bone metabolism, ATF4 critically affects the survival, proliferation, and terminal differentiation of osteoblast [56–59]. Mutations of RSK2, which can phosphorylate ATF4, causes the Coffin-Lowry syndrome, with its characteristic skeletal disorders [60]. ATF4 can additionally promote the formation of osteoprogenitors and induce osteoblast differentiation by upregulating the protein level of *β*-catenin in the Wnt/*β*-catenin signaling pathway [61], an extremely important pathway controlling bone mass [62, 63]. ATF4 is an upstream transcriptional activator of OSX [56], which is a key factor required for osteoblast differentiation and bone formation [64–66]. This upregulation involves interactions of ATF4 with a specific enhancer sequence in the Osterix promoter [56]. Osteocalcin (OCN), a specific osteoblast marker in late-stage differentiation, is a transcriptional target of ATF4, and ATF4 knockout mice showed a distinguished decrease in the expression level of OCN and bone mass formation [67]. ATF4 interacts with Runx2 and enriches the promoter activity of OCN [68–70]. Under endoplasmic reticulum stress, the PERK-eIF2*α*-ATF4 pathway is activated and further influences osteodifferentiation by targeting OCN [71].

These studies suggest a pivotal role of ATF4 in promoting osteoblast differentiation. Our data showed that miR-214 suppressed hPDLSC osteogenic differentiation (Figures 5(a) and 5(b)), and the protein level of ATF4, its target gene, was decreased (Figure 5(d)). The mRNA levels of RUNX2 and OCN, two osteoblast markers of osteodifferentiation, also decreased significantly (Figure 5(c)). Combined with the results of previous studies, we identified a new pathway in hPDLSC osteogenesis in which miR-214 suppressed ATF4 protein expression and accordingly repressed activation of OCN by decreasing the expression of RUNX2 (Figures 5(a) and 5(b)), thus downregulating osteogenesis.

## 5. Conclusions

In summary, our data add a new layer of regulation to osteogenic differentiation from hPDLSCs by miRNAs and indicate that miR-214 suppresses osteogenic differentiation of hPDLSCs by targeting ATF4. Taken together, these studies provide insight into how to achieve better osteogenic differentiation of hPDLSCs for use in tissue engineering.

## Supplementary Material

Figure S1. Characteristics of human periodontal ligament stem cells (hPDLSCs). Related to Figure 1. (a) CFU-F assays of 3 men and 3 women selected from the donors according to comparable age. (b) Adipogenic induction was verified by increased expression of LPL and PPAR*γ* in mRNA level. ∗∗∗*p* < 0.001.

## Figures and Tables

**Table 1:** Primer sequences used in quantitative real-time reverse transcription polymerase chain reaction.

Gene target	Sequence
ALP	Forward: 5′-TTCAAACCGAGATACAAGCACT-3′ Reverse: 5′-GGGCCAGACCAAAGATAGAG-3′
RUNX2	Forward: 5′-TGGTTACTGTCATGGCGGGTA-3′ Reverse: 5′-TCTCAGATCGTTGAACCTTGCTA-3′
OCN	Forward: 5′-GCAGCCACCGAGACACCAT-3′ Reverse: 5′-AGAGCGACACCCTAGACCG-3′
GAPDH	Forward: 5′-AGGTCGGAGTCAACGGATTTG-3′ Reverse: 5′-AGGCTGTTGTCATACTTCTCAT-3′
LPL	Forward: 5′-TCATTCCCGGAGTAGCAGAGT-3′ Reverse: 5′-GGCCACAAGTTTTGGCACC-3′
PPAR*γ*	Forward: 5′-ATGGTGGACACGGAAAGCC-3′ Reverse: 5′-CGATGGATTGCGAAATCTCTTGG-3′
U6	Forward: 5′-CGCTTCACGAATTTGCGTGTCAT-3′ Reverse: 5′-GCTTCGGCAGCACATATACTAAAAT-3′
