# Supplementary material for: MicroRNA-214 Suppresses Osteogenic Differentiation of Human Periodontal Ligament Stem Cells by Targeting ATF4
Source: Stem Cells Int. 2017 Oct 29;2017:3028647. doi: 10.1155/2017/3028647 (PMC5682087; doi:10.1155/2017/3028647)
Supplement: Supplementary file 1 — Figure S1. Characteristics of human periodontal ligament stem cells (hPDLSCs). Related to Figure 1. (a) CFU-F assays of 3 men and 3 women selected from the donors according to comparable age. (b) Adipogenic induction was verified by increased expression of LPL and PPARγ in mRNA level. ∗∗∗p < 0.001. [file 3028647.f1.pdf]

Supplemental Figure

a

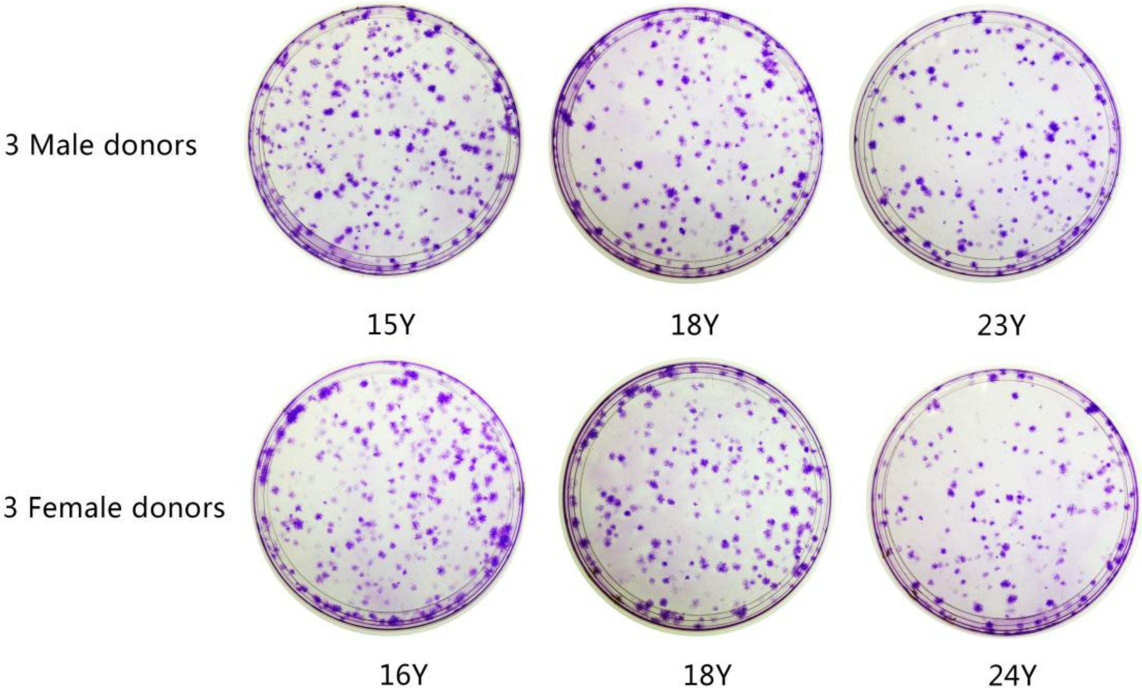

b

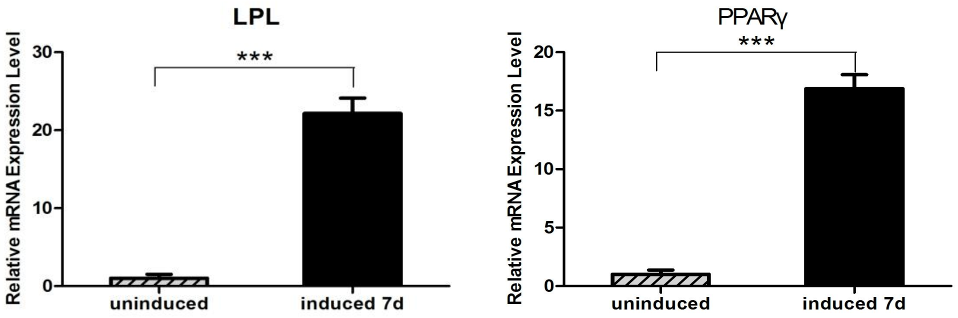

Figure S1. Characteristics of human periodontal ligament stem cells (hPDLSCs).

Related to Figure 1.

(a) CFU-F assays of 3 men and 3 women selected from the donors according to comparable age.

(b) Adipogenic induction was verified by increased expression of LPL and PPARγ in mRNA level. ***$p<0.001$.
